# Prediction of Xe/Kr Separation in Metal-Organic Frameworks by a Precursor-Based Neural Network Synergistic with a Polarizable Adsorbate Model

**DOI:** 10.3390/molecules28217367

**Published:** 2023-10-31

**Authors:** Zewei Liu, Qibin Xia, Bichun Huang, Hao Yi, Jian Yan, Xin Chen, Feng Xu, Hongxia Xi

**Affiliations:** 1 School of Environmental and Chemical Engineering, Foshan University, Foshan 528000, China; liuzewei@fosu.edu.cn (Z.L.); yanjian@fosu.edu.cn (J.Y.); chenxin@fosu.edu.cn (X.C.); 2 School of Chemistry and Chemical Engineering, South China University of Technology, Guangzhou 510640, China; qbxia@scut.edu.cn; 3 School of Environment and Energy, South China University of Technology, Guangzhou Higher Education Mega Centre, Guangzhou 510006, China; cebhuang@scut.edu.cn; 4 South China Institute of Environmental Sciences, Ministry of Environmental Protection, Guangzhou 510655, China; yihao@scies.org; 5 Guangdong Provincial Key Laboratory of Atmospheric Environment and Pollution Control, South China University of Technology, Guangzhou Higher Education Mega Centre, Guangzhou 510006, China

**Keywords:** Xe/Kr, polarizable model, MOFs, precursor-based BPNN model, physical parameter

## Abstract

Adsorption and separation of Xe/Kr are significant for making high-density nuclear energy environmentally friendly and for meeting the requirements of the gas industry. Enhancing the accuracy of the adsorbate model for describing the adsorption behaviors of Xe and Kr in MOFs and the efficiency of the model for predicting the separation potential (SP) value of Xe/Kr separation in MOFs helps in searching for promising MOFs for Xe/Kr adsorption and separation within a short time and at a low cost. In this work, polarizable and transferable models for mimic Xe and Kr adsorption behaviors in MOFs were constructed. Using these models, SP values of 38 MOFs at various temperatures and pressures were calculated. An optimal neural network model called BPNN-SP was designed to predict SP value based on physical parameters of metal center (electronegativity and radius) and organic linker (three-dimensional size and polarizability) combined with temperature and pressure. The regression coefficient value of the BPNN-SP model for each data set is higher than 0.995. MAE, MBE, and RMSE of BPNN-SP are only 0.331, −0.002, and 0.505 mmol/g, respectively. Finally, BPNN-SP was validated by experiment data from six MOFs. The transferable adsorbate model combined with the BPNN-SP model would highly improve the efficiency for designing MOFs with high performance for Xe/Kr adsorption and separation.

## 1. Introduction

In 2050, high-density nuclear energy is expected to account for a minimum of 15% of China’s primary energy resources, serving as a sustainable alternative to fossil fuels. To achieve this objective, it is crucial to address the radiation emissions resulting from the reprocessing of nuclear waste, specifically the Xe/Kr mixture, during nuclear electricity generation. The longest half-life period for the isotope elements Xe (Xe^127^) and Kr (Kr^85^) are 36.4 days and 10.73 years, respectively. By effectively capturing the Xe/Kr mixture produced during reprocessing, high-density nuclear energy can become more environmentally friendly. Furthermore, the properties of xenon (Xe) and krypton (Kr), including their non-flammable inert nature and low boiling points, have led to their widespread application in various industries, such as insulating gases, lighting, imaging, tracer applications, and leak detection, etc. [1,2,3,4]. For instance, Xe is a useful propellant in ion propulsion engines, while Kr can be applied as electric light sources, gas lasers, and in plasma flows. Considering the above, it is significant to develop an effective technology to obtain Xe/Kr mixture or highly purified species, which can not only reduce the radioactive gas emission but also satisfy industrial requirements. However, the inert nature and the extremely low concentration of Xe and Kr (Xe: 0.087 ppmv, Kr: 1.14 ppmv) in the air make it difficult to capture highly purified Xe or Kr. One of the main sources for Xe/Kr mixture, with an 80:20 molar ratio for Kr to Xe, is from the side product stream from a series of steps in producing nitrogen and oxygen. Further treatment is needed for generating highly purified Xe or Kr. Compared to traditional cryogenic distillation, pressure swing adsorption (PSA) can be an alternative method for the adsorption and separation of binary gas mixture due to its high efficiency, energy, and cost saving. Therefore, highly effective PSA technology is indispensable to promote the nuclear electricity energy generation and meet the industrial requirements.

The performance of PSA technology is mainly determined by the adsorbent. In comparison to traditional nanoporous adsorbents, metal-organic frameworks (MOFs), which are assembled by organic linkers and metal nodes, have been widely used throughout several applications, especially in the field of gas adsorption and separation, owing to their high surface area, large pore volume, tunable structure, and rich functionality [5,6,7,8,9,10]. It has been confirmed that MOFs can be a promising platform for adsorption and separation of Xe/Kr mixture with considerable capacity and selectivity [11,12,13,14,15,16,17,18]. PCN-14 [17] exhibited remarkable adsorption capacity of Xe, reaching up to 7.2 mmol/g at 1 bar and 292 K. Meanwhile, a Co-based MOF [18] was reported to possess an impressive selectivity for Xe/Kr mixture, with a value of 69.7. It is worth noting that both adsorption capacity and selectivity of Xe/Kr mixture in MOFs surpass those of traditional materials. Nevertheless, it is a rare occurrence to find MOFs that possess both high adsorption uptake and selectivity of Xe/Kr mixture. To evaluate the adsorption and separation performance of binary gas mixtures in MOFs, certain criteria have been established. One of them is separation potential (SP), which serves as an indicator to reflect the fixed bed breakthrough process. It takes into consideration both adsorption capacity and selectivity in a comprehensive way [19,20]. Nonetheless, the diversity of MOFs presents a challenge when it comes to efficiently and cost-effectively identifying promising MOFs with large SP value for Xe/Kr mixture. Traditional methods for such screening demand a large amount of experimental data, incurring significant costs and time constraints. In view of searching for promising MOFs within a short time and at a low cost, it becomes imperative to unravel the intricate relationship between MOF structures and their performance. A potential solution to this issue involves the application of molecular simulation, high-throughput screening, and the development of an artificial neural network (ANN) model. These approaches offer a more efficient shortcut for addressing this challenge.

Grand Canonical Monte Carlo (GCMC) simulation has been utilized to delve into the intricacies of the adsorption and separation mechanisms of Xe/Kr in MOFs [17,21,22,23,24,25]. However, the traditional models for description of Xe and Kr have poor performance to mimic Xe and Kr adsorption behaviors in some MOFs due to the flaw of the model in expressing adsorbent–adsorbate interaction induced by the polarizability of the adsorbate and the electric field of the adsorbent [17,26]. Previous reports employed correction from density functional theory (DFT) to enhance the accuracy of the model for describing adsorption behaviors of Xe and Kr in MOFs [27,28]. Such correction technology has high precision on specific or several MOFs analogues, but when it comes to the high-throughput screening, this method would consume a lot of time when generating various Lennard-Jones (LJ) parameters because the correction varies with each MOF. Therefore, to develop a universal model for studying the adsorption and separation behaviors of Xe/Kr mixture in MOFs by dealing with the induced polarization issue is significant.

Combined with high-throughput technology, artificial neural network is an effective tool for investigating the correlation between MOF structures and their adsorption performances [23,29,30,31,32,33]. As demonstrated by Cory M. Simon and colleagues, the most favorable environments within MOFs for Xe/Kr separation are characterized by pocket-like, ring-like, tube-like, and cage-like structures. This conclusion was drawn by a screening for Xe/Kr separation performances of 670,000 predicted nanoporous materials including MOFs. They constructed a bond between selectivity and pore size by mathematical models [33]. Seda Keskin et al. investigated 115 different MOFs at their adsorption and separation performance of noble gas mixtures using molecular simulation. Results suggested that MOFs have pore sizes, surface areas, and porosities in the range of 4.3–6.8 Å, 150–1000 m^2^/g, and 0.37–0.58, respectively [23]. On the strength of textural properties such as pore limiting diameter, largest cavity diameter, surface area, etc., previous methodologies (called product-based prediction methodology) could predict adsorption and separation performance of MOFs or give theoretical guidance for designing MOFs with high performance at specific temperatures and pressures with high accuracy [19,32,34,35,36]. However, such product-based methodology can only predict performance based on textural properties of synthesized or designed MOFs, which is a flaw in terms of saving time and in terms of economic cost. What is more, most of previous prediction models were generated from the GCMC simulation using the traditional model for Xe and Kr. Therefore, there is still room for improving the accuracy of the prediction model as long as it is served by a new universally polarized model. Herein, a prediction methodology (called precursor-based prediction methodology) that can only use physical parameters of MOFs precursors (such as the three-dimensional size of the organic linker with radius and the electronegativity of the metal center) to predict adsorption and separation performance of Xe/Kr mixture in MOFs is proposed, which can help to reduce the time and economic cost for studying the structure–activity relationship and for designing MOFs with excellent performance.

In this study, GCMC simulation was performed to establish universally polarized models for Xe and Kr, which were tested by seven MOFs, including MOFs with various topologies, organic linkers, and metal centers. DFT, high-throughput screening, and ANN modeling were combined to delve into the structure–activity relationship between structures of 38 MOFs and their performance in terms of adsorption and separation of an Xe/Kr mixture at various temperatures (P = 278 K, 288 K, 298 K, 308 K, and 318 K) and pressures (T = 0.1 bar, 0.4 bar, 1 bar, 10 bar, 35 bar, and 50 bar). Finally, the optimal ANN model was validated by experiment data from six MOFs.

## 2. Results and Discussion

### 2.1. Adsorption Isotherms of Xe and Kr

**Figure 1:**
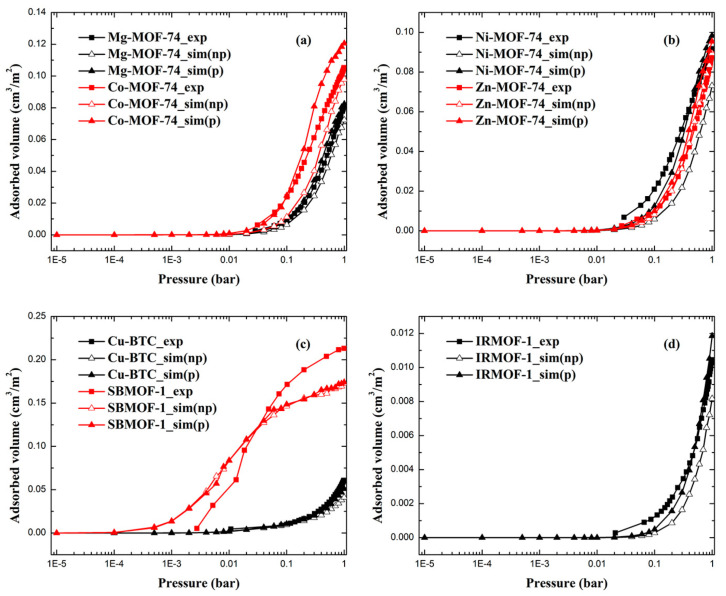
The comparison between simulated and experimental adsorption isotherms of Xe in (**a**) Mg-MOF-74, Co-MOF-74; (**b**) Ni-MOF-74, Zn-MOF-74; (**c**) Cu-BTC, SBMOF-1 and (**d**) IRMOF-1 (np: model without polarization, p: model with polarization).

**Figure 2:**
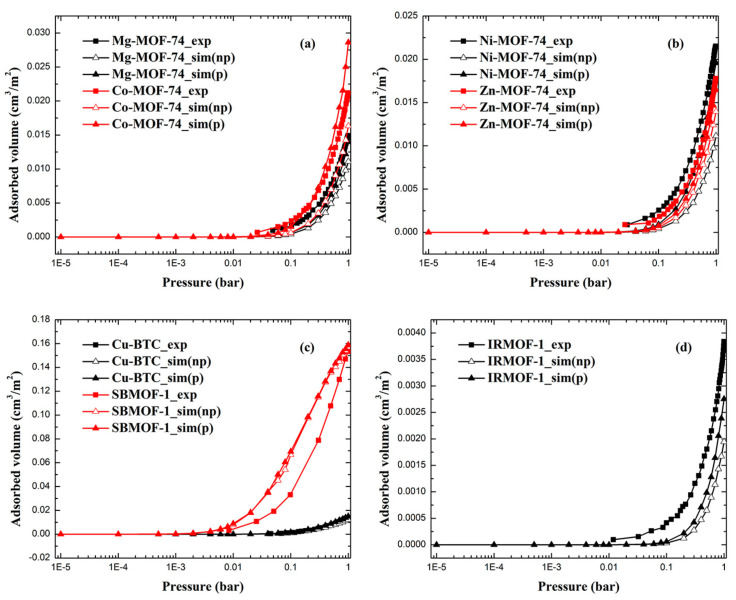
The comparison between simulated and experimental adsorption isotherms of Kr in (**a**) Mg-MOF-74, Co-MOF-74; (**b**) Ni-MOF-74, Zn-MOF-74; (**c**) Cu-BTC, SBMOF-1 and (**d**) IRMOF-1 (np: model without polarization, p: model with polarization).

The comparison of simulated and experimental adsorption isotherms for Xe and Kr is displayed in Figure 1 and Figure 2. MOFs including M-MOF-74 (M = Mg, Co, Ni, Zn), Cu-BTC, IRMOF-1, and SBMOF-1 were utilized in this study to test the accuracy of the polarizable model and its transferability. Among these MOFs, M-MOF-74 and Cu-BTC are MOFs with open metal sites, while IRMOF-1 and SBMOF-1 are MOFs without open metal sites. In addition, these MOFs consist of various pore size distributions, metal centers, and organic linkers, indicating the diversity of such sets of MOFs. In view of eliminating the effect of difference between theoretical and experimental surface area on the adsorption uptake, the unit of adsorption uptake was cm^3^/m^2^, which was transferred from cm^3^/g (volumetric uptake) divided by m^2^/g (surface area). As can be seen in Figure 1 and Figure 2, most of the simulated Xe and Kr adsorption isotherms generated by the model without polarization underestimate the adsorption uptake compared with experiment data. The consideration of polarization did enhance adsorbate–adsorbent interaction due to the extra polarization energy, suggesting that adsorption behaviors of Xe and Kr can be restored using the polarization model. All adsorption isotherms of both Xe and Kr in MOFs, except for Xe in SBMOF-1, are in the same shape compared with experimental ones. Of note, not all adsorption uptakes of Xe and Kr can be obviously increased by the impulse of the polarization effect, especially for MOFs without open metal sites such as SBMOF-1 and IRMOF-1, because the contribution of the polarization energy to adsorption uptake is mainly determined by the distribution of the electronic cloud of the metal center. For Xe and Kr adsorption in SBMOF-1, the polarization model can maintain a similar accuracy for the prediction of adsorption isotherms because of almost equal adsorption uptake predicted from models with and without polarization effect.

In order to figure out the accuracy of the model with or without polarization consideration, two types of root-mean-squared errors (RMSE-I and RMSE-II) were generated using the following equations.

(1) $$RMSE-I=\sqrt{\frac{{\left(N_{ads}-N_{exp}\right)}^{2}}{n}}$$

(2) $$RMSE-II=\sqrt{\frac{{\left(\frac{N_{ads}-N_{exp}}{N_{exp}}\right)}^{2}}{n}}$$

where N_ads_ and N_exp_ are adsorption uptake of Xe or Kr from simulation and experiment, respectively. n is the number of data points from the experimental isotherm.

RMSE-I and RMSE-II value for Xe and Kr adsorption in MOFs are listed in Table 1 and Table 2, respectively. As can be seen in Table 1, the RMSE-I value of the traditional np model was reduced from 0.013 cm^3^/m^2^ to 0.010 cm^3^/m^2^, while the RMSE-II value was reduced from 28% to 19%. For MOFs like Ni-MOF-74 and IRMOF-1, polarization consideration can reduce the RMSE-II value by 50%. Since the polarizability of Kr is much smaller than that of Xe, the effect of polarization consideration on adsorption uptake of Kr should be less than that of Xe, especially in MOFs without open metal sites. However, the polarization model can also get better at mimicking the adsorption behavior of Kr in MOFs. As shown in Table 2, the RMSE-I value of the traditional np model was reduced from 0.005 cm^3^/m^2^ to 0.004 cm^3^/m^2^, while the RMSE-II value was reduced from 38% to 27%. From the above, we can conclude that the polarization model can well predict adsorption uptakes of Xe and Kr in MOFs with and without open metal sites.

### 2.2. Selectivity of Xe/Kr Mixture

**Figure 3:**
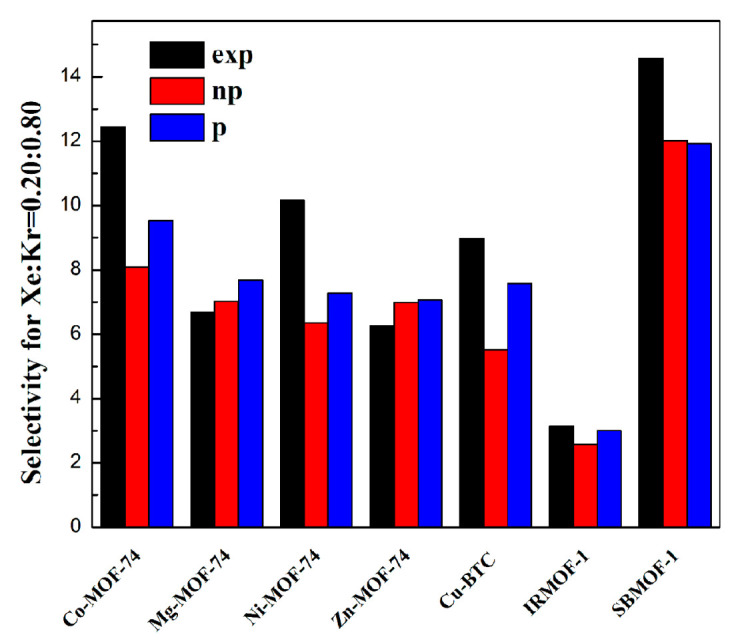
Comparison of selectivities for Xe/Kr mixture in tested MOFs obtained by experiment and models with and without polarization.

In order to do a comprehensive study on the polarization model, RMSE values (as shown in Table 3) for selectivity of Xe/Kr mixture in MOFs were also tested. Comparisons of selectivities for Xe/Kr mixture in tested MOFs generated from an experiment using IAST and models with or without polarization and GCMC simulation are shown in Figure 3. The reason for using selectivity calculated from a GCMC simulation is that a GCMC simulation can be more accurate in mimicking the adsorbate–adsorbent interaction, which can be influenced by polarization interaction in real time, while IAST cannot well describe such polarization interaction. Futures studies could focus on performances of MOFs at various pressures even higher than 1 bar, using GCMC simulation with polarization consideration to predict selectivity of Xe/Kr mixture in a MOF, which would make the prediction more accurate. As can be seen in Figure 3, simulation without polarization consideration obviously underestimated selectivity of Xe/Kr in some unsaturated MOFs, including Co-MOF-74, Ni-MOF-74, and Cu-BTC. Simulation with polarization consideration could reduce the difference between experiment and simulation for these three saturated MOFs. As can be seen in Table 3, the RMSE-I value for prediction of selectivity was reduced from 2.75 to 1.06, while the RMSE-II value was reduced from 26% to 18%. It can be safely concluded that the polarization model is a transferrable model for describing adsorption and separation performance of Xe/Kr in MOFs well, including those that consist of various metal centers and organic linkers.

### 2.3. ANN Model

In order to clarify the relationship between the physical parameters of MOF precursors (metal center and organic linker) and the separation potential of the Xe/Kr mixture in a MOF, a new BPNN (back propagation neuron network) model was designed following various trial and error tests. Herein, inputs consist of physical parameters, temperature, and pressure, while the separation potential of the Xe/Kr mixture was set as output data. Firstly, mean absolute error (MAE), mean bias error (MBE), root-mean-squared error (RMSE), and regression coefficient (R) were calculated to compare performances of different models. An optimal model should have the lowest MAE, MBE, and RMSE value with the highest R value, and these were calculated by the following equations.

(3) $$MAE=\frac{1}{N}\sum_{i=1}^{N}\left|{targets}_{i}-{outputs}_{i}\right|$$

(4) $$MBE=\frac{1}{N}\sum_{i=1}^{N}\left({targets}_{i}-{outputs}_{i}\right)$$

(5) $$RMSE=\sqrt{\frac{1}{N}\sum_{i=1}^{N}{\left({targets}_{i}-{outputs}_{i}\right)}^{2}}$$

where targets and outputs refer to the SP value from the GCMC simulation and the prediction by the ANN model.

**Figure 4:**
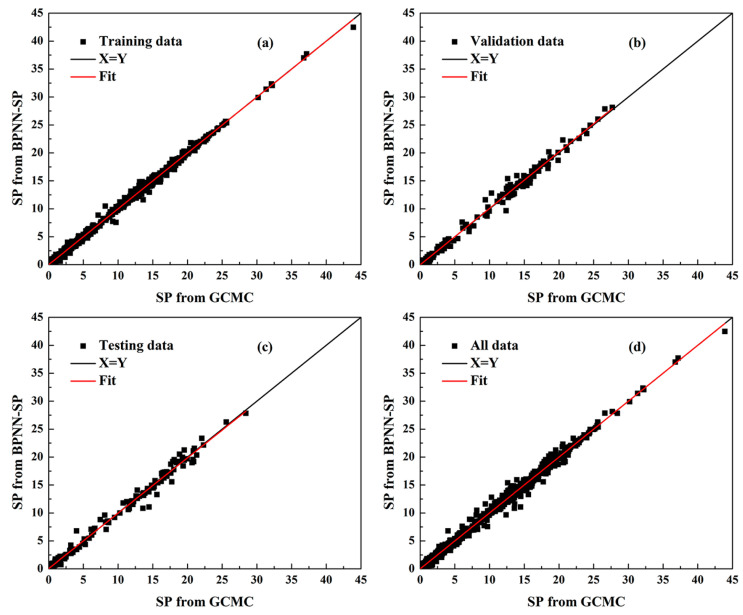
Comparison of SP value from GCMC and BPNN-SP for (**a**) Training data; (**b**) Validation data; (**c**) Testing data, and (**d**) All data.

**Figure 5:**
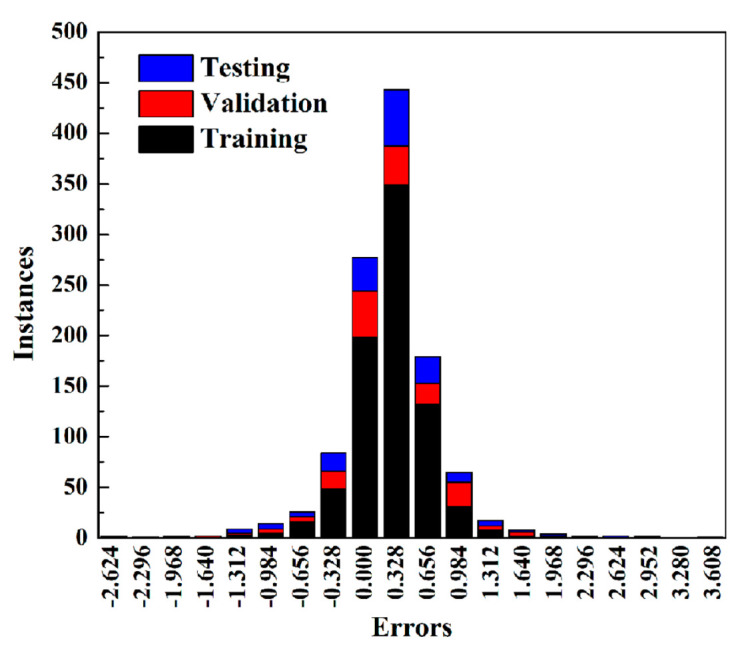
Error histograms of numbers of each target from the BPNN-SP model.

All values for various models are listed in Table 4. Overall, R values for each data set from each model were all larger than 0.96, indicating that the BPNN model appears tailor-made for predicting SP value based on physical parameters of MOF precursors, temperature and pressure with strong associativity. It is obvious that model No. 14 (denoted as BPNN-SP) is the best one with the lowest MAE, MBE, and RMSE values accompanied by the highest R value, which is higher than 0.995 for each data set. It is toted that the MBE value of the BPNN-SP model is only −0.002 mmol/g, while MAE and RMSE are the lowest ones, suggesting that the BPNN-SP model is a well-designed model for predicting the separation factor of Xe/Kr mixture in MOFs at various temperatures and pressures based on the physical parameters of the MOF precursors. In addition, Figure 4 gives an insight on the correlationship between SP values predicted by the BPNN-SP model and those simulated through the GCMC method for each data set. As can be seen in Figure 4, results of SP values from GCMC range from 0 to 42.5 mmol/g. Combining a high R value (larger than 0.995) from Table 4 for each data set, Figure 4 shows that the fit line and diagonal line was highly correlated for each data set. The error histograms for each target from the BPNN-SP model shown in Figure 5 indicate that the maximum error among all data is only 3.608 mmol/g, accounting for just one output from the testing data set. It must be pointed out that those relatively high errors are generated from data with high SP values. Therefore, those relatively high errors virtually also refer to low errors in percentage. What is more, the rate of errors settles in the range of −0.328 mmol/g to 0.328 mmol/g and can go as high as 65.0%. Even better is that about 94.3% of errors stay inside the range of −0.984 mmol/g to 0.984 mmol/g. Finally, the BPNN-SP model can well predict separation potential for Xe/Kr mixture in MOFs at various temperatures and pressures based on the physical parameters of the MOF precursors. The weights and biases of the BPNN-SP model for connection of each neuron and between different layers are listed in the Supplementary Materials S1.

### 2.4. Sensitivity Analysis

To determine the proportionate influence of each variable within the BPNN-SP model for predicting SP value of Xe/Kr mixture in MOFs, a commonly employed sensitivity analysis approach known as the PaD method was utilized [37,38,39,40]. In comparison to alternative sensitivity analysis techniques, the PaD method in this study offers greater stability and reliability with clear contribution for input variables. The core concept behind the PaD method involves computing partial derivates of the output, generated by input variables, taking into account the relative weights and biases in each layer. Herein, the partial derivates of output, associated with each variable, can be calculated using

(6) $$\left(\frac{\partial N_{o}}{\partial x_{i}}\right)=\sum_{k=1}^{n}\sum_{j=1}^{m}\left[W_{k,l}(1-N_{k}^{2})W_{j,k}(1-N_{j}^{2})W_{i,j}\right]$$

where N_o_ is the output derived from the last transfer function, namely the separation potential for Xe/Kr mixture predicted by the BPNN-SP model. Both m and n are 20 and 10, which point to the number of neurons in two hidden layers. W_ij_, W_jk_, and W_kl_ refer to weight linked to each neuron in each layer. The relative contribution of each input variable was generated by the sum of the squared partial derivatives (SSD_i_), which was calculated by

(7) $${SSD}_{i}=\sum_{\gamma =1}^{Z}{\left(\frac{\partial N_{o}}{\partial x_{i}}\right)}_{\gamma }^{2}$$

where Z is the number of all data, which is equal to 1140, and γ is the index of data point. Finally, the percentage contribution of each input variable was generated using

(8) $$contribution\;of\;ith\;variable\;\left(\%\right)=\frac{{SSD}_{i}}{\sum_{i=1}^{n}{SSD}_{i}}\times 100$$

**Figure 6:**
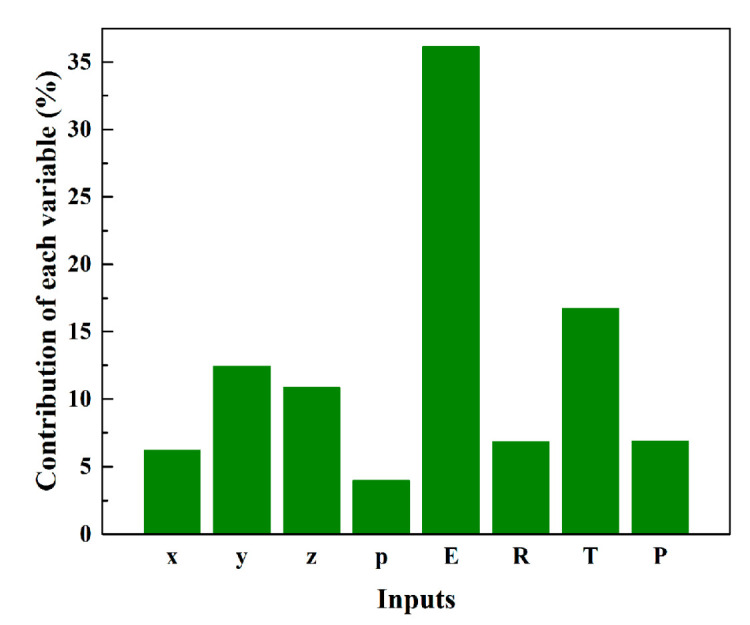
Percentage contribution of each variable for predicting SP value. (x sizex, y: sizey, z: sizez, p: polarizability, E: electronegativity, R: radius, T: temperature, P: pressure).

The percentage contribution of each variable for predicting SP value is shown in Figure 6. As shown in Figure 6, percentage contribution of electronegativity (E, 36.12%) is much larger than other variables, implying the important role that induced energy plays in the adsorption and separation of Xe/Kr. The expression for calculating induced energy suggests that the energy could be more easily influenced by the electric field because of the secondary power. What is more, electronegativity can indirectly reflect the strength of the electric field of the metal center. This is also the reason that the insertion of induced energy can obviously enhance the accuracy of the traditional model. The radius of the metal center refers to the exposure of the metal center to adsorbates. This is to say, a larger radius can increase the contact environment for the metal center to interact with adsorbates. However, the strength of the metal–adsorbate interaction is firstly decided by the electronegativity of the metal center. Therefore, the percentage contribution of the radius is obviously smaller than that of electronegativity. In terms of physical parameters of the organic linker, all of them get comparable percentage contribution, which follow the order of sizey > sizez > sizex > polarizability. It suggests that three-dimensional size is more significant than the polarizability of the organic linker for SP value. Combined with the radius of the metal center, three-dimensional size of the organic linker can be a descriptor for the pore size of a MOF. More importantly, the strength of the linker–adsorbate interaction is influenced to some degree by the pore size in terms of noble gas adsorption in MOFs. A small pore size can promote the synergistic effect from various organic linkers in a small cage. Then the polarizability of the organic linker is the second important factor to influence the linker–adsorbate interaction. The strength of the polarizability refers to the concentration degree of electron density of the organic linker, which can also reflect the electric field that the organic linker can afford for the polarized noble gas molecule. It can certainly be concluded that the metal center plays a more significant role in adsorption and separation of Xe/Kr mixture in MOFs. The diffusivity and adsorption uptake of the adsorbate varies at different temperatures and pressures, which can directly influence the SP value of a MOF by the difference caused by various T and P on Xe and Kr. Therefore, the influence of temperature and pressure cannot be ignored.

### 2.5. Validation of ANN Model

Experiment data from six MOFs (NOTT-100, NOTT-101, NOTT-102, NOTT-103, PCN-14, and UiO-66), which consist of various organic linkers, metal centers, and topologies from previous reports [17,41], were selected to validate the reliability and generalizability of the BPNN-SP model. Since pressures of experimental gas adsorption isotherms for these selected MOFs only lay between 0 to 1 bar, only comparisons between SP values generated from experiment data and the BPNN-SP model at 0.1, 0.4, and 1 bar were made. As can be seen in Table 5, SP values from the BPNN-SP are similar to those from experiment data for related MOFs. It is noted that MOFs selected for validation of the BPNN-SP model possess various metal centers, organic linkers, and topologies; the examination of the reliability of the BPNN-SP model in this work is comprehensive. In addition, the RMSE value of the SP values predicted from the BPNN-SP model, compared with that from experiment data, is only 0.248 mmol/g. It is worth noting that predicted SP values for pressure at 0.1 bar have a relatively larger deviation compared with those at 0.4 bar and 1 bar. The main reason for this phenomenon is that uptakes of both Xe and Kr at 0.1 bar are so small that adsorbate molecules cannot adequately interact with an adsorption site, such as the metal center or the organic linker. Another reason is that the simulated model for a MOF is an ideal model so that the density of adsorption site in the simulated model may be richer than that in the experimental model. These two reasons result in the minification on the effect of polarizability and electronegativity on the adsorption and separation of Xe/Kr. As the uptake of Xe or Kr increases with the increasing pressure, the degree of involvement of each physical parameter for MOFs precursors increases because adsorbates gradually occupy adsorption sites and the pore of MOFs. It can be safely concluded that predicted SP values will be close to experimental ones at higher pressures. Therefore, the BPNN-SP model trained in this work should be a reliable and precise model for predicting SP values for adsorption and separation of Xe/Kr in MOFs based on the physical parameters of MOFs precursors.

## 3. Methods

### 3.1. Model Construction and Structure Characterization

Models of existing MOFs for testing the polarized model of Xe and Kr were from Cambridge Crystal Data Centre (CCDC). The theoretical MOFs models for further study of the structure–activity relationship were derived from existing MOFs by replacing the metal center. The MOFs set in this study consists of M-BTC (M = Cu, Co, Fe, Mo, Ti, Ru), M-TDPAT (M = Cu, Co, Fe, Mo, Ti, Ru), M-IRMOF-1 (M = Zn, Fe, Cd, Ni), M-IRMOF-6 (M = Zn, Fe, Cd, Ni), M-IRMOF-7 (M = Zn, Fe, Cd, Ni), M-IRMOF-10 (M = Zn, Fe, Cd, Ni), M-MOF-74 (M = Co, Cu, Fe, Mg, Mn, Ni, Ti, V, Zn), and Ca-SBMOF-1. Note that all MOFs in this study are experimentally existing or theoretically feasible according to previous reports [16,28,42,43,44,45]. All MOFs models were then optimized using Forcite Module from Materials Studio 7.0 [46] with ultrafine quality, which has been widely applied in optimization of a MOF model [47,48,49,50]. The convergence criteria for energy are 1 × 10^−5^ kcal/mol, force is 0.5 × 10^−5^ kcal/mol/Å, and displacement is 1.0 × 10^−6^ Å. All optimized structures remained regular and intact. To determine physical parameters, such as three-dimensional size and polarizability of the organic linker, the organic linker should be cut from the MOF unit cell. To maintain the integrity of the ligand’s structure, hydrogen atoms were added at the points where the structure was cut. Subsequently, the organic linker underwent optimization using Gaussian 09 [51], employing the m06/6-311 + g(d,p) basis set. Simultaneously, polarizability of the linker was calculated using the same basis set. Multiwfn 3.8 [52] was employed to generate three-dimensional size, denoted as sizex, sizey and sizez for the optimized organic linker. Herein, sizex represents the length of the organic linker, while sizey corresponds to the direction parallel to the carboxyl group. Sizez is vertical to the xy plane, providing insight into the thickness of the organic linker. Followed by sizey and sizez, the largest value among the three-dimensional size points to sizex for an organic linker with irregular shape.

### 3.2. Simulation Details

Grand Canonical Monte Carlo (GCMC) simulation was conducted in the RASPA 2.0 [53]. In the simulation, the adsorbent-adsorbate and adsorbate-adsorbate Van der Waals interaction was descried using Lennard-Jones potential [54]. The crosses-interaction was expressed by Lorentz–Berthelot mixing rules.

(9) $$U_{ij}=4\varepsilon_{ij}[{\left(\frac{\sigma_{ij}}{r_{ij}}\right)}^{12}-{\left(\frac{\sigma_{ij}}{r_{ij}}\right)}^{6}]$$

(10) $$\varepsilon_{ij}={\left(\varepsilon_{i}\times \varepsilon_{j}\right)}^{\frac{1}{2}}$$

(11) $$\sigma_{ij}=\frac{1}{2}(\sigma_{i}+\sigma_{j})$$

where ε and σ are energy well depth and Van der Waals radius, respectively. r_ij_ refers to the distance between atom i and j.

Combined with universal force field (UFF) [55] for metal atom, Dreiding force field [56] for non-metal atom was used to describe the atom from the adsorbent. Xe and Kr were both considered as single-atom sites with force field parameters from previous reports. Details of force field parameters for all atoms are listed in Table S1 in Supplementary Materials S2.

Besides VDW interaction, there is an induction energy (U_ind_) induced by the polarized effect on the adsorbate from the electric field of the adsorbent. Only polarization between adsorbent and adsorbate was considered. The field was described by charge from EQEq calculation. In this simulation, induced dipoles μ_i_ were calculated as follows [57]:

(12) $$\mu_{i}=\alpha_{i}\cdot E_{i}^{0}$$

where α_i_ is the static dipole polarizability of the adsorbate molecule, $E_{i}^{0}$ refers to the electric field created from the interaction site i of the framework. α_i_ for Xe and Kr in this work were set as 1.5 Å^3^ and 0.924 Å^3^, respectively.

The induction energy U_ind_ can be generated by the accumulation of the static electric field on the interaction site pointing to the static dipole polarizabilities [57].

(13) $$U_{ind}=-\frac{1}{2}\sum_{i=1}^{n}\alpha_{i}\cdot {\left|E_{i}^{0}\right|}^{2}$$

where n is the total amount of interaction sites of the moved adsorbate molecule. The model with and without polarization effect are denoted as p and np model, respectively. It is noted that the calculation of induction energy neglects the back-polarization.

The cutoff for the simulation is 12.8 Å, so lattices of all MOFs cells should be kept at least 25.6 Å. Adsorption uptakes for pure Xe and Kr at various temperatures and pressures were calculated. The Peng–Robinson equation of state was employed to translate fugacity into pressure [58]. To facilitate a comparison between simulations and experimental data, the absolute uptake (N_abs_) was transformed into excess uptake (N_exc_) by

(14) $$N_{exc}=N_{abs}-\rho_{g}V$$

where ρ_g_ and V are the gas density under specific condition and the pore volume of the framework determined by applying the ideal gas law [59].

The Dual-Sites Langmuir–Freundlich (DSLF) model was used to fit the simulated and experimental pure gas adsorption isotherms for further calculation about selectivities of Xe/Kr mixture in MOFs.

(15) $$Q=q_{m,1}\times \frac{b_{1}p^{n_{1}}}{1+b_{1}p^{n_{1}}}+q_{m,2}\times \frac{b_{2}p^{n_{2}}}{1+b_{2}p^{n_{2}}}$$

where Q represents the equilibrium uptake at pressure p for the bulk gas, q_m,i_ signifies the saturation loading at site i, b_i_ stands for the adsorption affinity coefficient of site i and n_i_ pertains to the deviation from an ideal homogenous surface.

With the fitting parameters from the DSLF model, Ideal Adsorbed Solution Theory (IAST) [60], which has been widely used to evaluate the selectivity of gas mixture in a MOF [5,61,62], was employed for generating selectivity of Xe/Kr mixture in MOFs. The selectivity was described as

(16) $$S_{ij}=\frac{x_{i}}{x_{j}}\frac{y_{j}}{y_{i}}$$

where x_i_ and x_j_ denote the molar fraction of component i and j within the adsorbed phase, while y_i_ and y_j_ indicate the molar fraction of component i and j in the gas phase.

SP, which can embody the potential of a MOF when used in fixed bed adsorption, was chosen to be the performance descriptor in this study.

(17) $$SP=N_{ads,Xe}\times \frac{y_{Kr}}{y_{Xe}}-N_{ads,Kr}$$

where N_ads,Xe_ and N_ads,Kr_ are adsorption uptake of Xe and Kr from the mixture in MOFs, respectively. y_Xe_ and y_Kr_ refer to molar fraction of Xe and Kr.

### 3.3. ANN Model

To establish the connection between MOFs and their SP values for Xe/Kr mixture, artificial neural network was employed. The objective was to develop an optimal model for predicting SP values for MOFs, utilizing textural characteristics of MOFs precursors, as well as temperature and pressure as inputs. The back propagation neural network (BPNN) model, a well-established ANN technology, has found extensive application in the analysis of the structure–activity relationship between MOFs and their performances [63,64,65,66].

**Figure 7:**
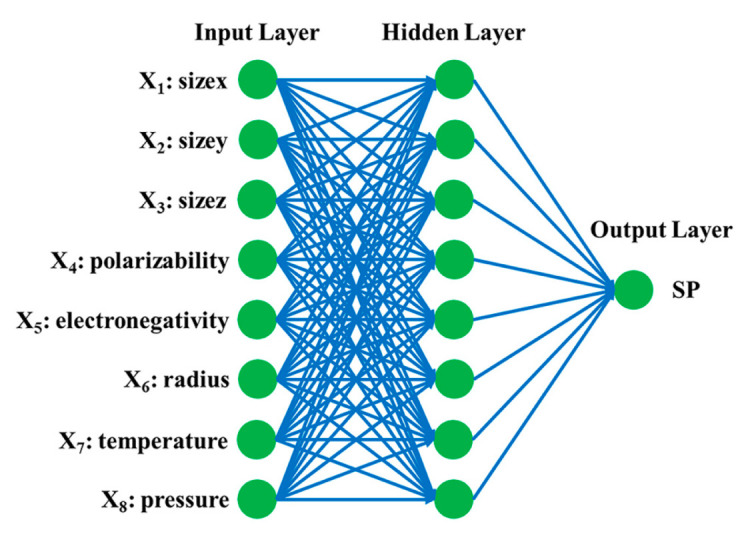
Schematic diagram of the BPNN model for prediction of SP value based on eight inputs.

Figure 7 shows the workflow of a typical BPNN model, which consists of an input layer comprising input data, one or more hidden layers comprising some neurons in each layer, and an output layer comprising output data. Levenberg−Marquardt optimization as the training function of the BPNN model used in this work is a faster method that can more easily avoid trapping to local minima compared with other optimization methods. Each layer was interlinked by transfer functions (TF_1_, TF_2_, and TF_3_), which are expressed as follows:

(18) $${TF}_{1}=\frac{1}{1+e^{-n}}$$

(19) $${TF}_{2}={TF}_{3}=\frac{2}{1+e^{\left(-2n\right)}}-1$$

where n is the output from the former layer.

Combined with transfer function, weight (W) and bias (B) were employed to enlarge the freedom of data distribution and adjust data to be approximately equal with target data. w is updated by

(20) $$dW=M\times {dW}_{prev}+(1-M)\times LR\times gW$$

where M is equal to 0.9 for momentum constant, W_prev_ refers to weight in previous training, LR is equal to 0.01 for learning rate, and gW is the random gradient for a weight.

With the changing W and B, data in hidden layer and output layer were transferred by transfer function through the following equation.

(21) $$Y={TF}_{3}\left[\sum_{k=1}^{l}\left({TF}_{2}\left[\sum_{j=1}^{k}\left({TF}_{1}\left[\sum_{i=1}^{j}X_{i}W_{ij}+B_{j}\right]\right)W_{jk}+B_{k}\right]\right)W_{kl}+B_{l}\right]$$

where i, j, k, and l are input layer, 1st hidden layer, 2nd hidden layer, and output layer, respectively. X_i_ is the input data.

On account of the efficiency and ease of handling, a widely used cross validation technique known as “hold-out” was implemented to prevent overtraining of the BPNN model during the modeling process. Within the training process, the data set was divided into three distinct subsets: training, validation, and testing data, with the respective proportions of 70%:15%:15%. All subsets were randomly extracted from the main dataset.

In this study, physical parameters, such as the three-dimensional size and the polarizability of organic linkers along with the radius and electronegativity of the metal center were considered in conjunction with varying temperature and pressure ranges as inputs, while SP values of Xe/Kr mixture in MOFs at each temperature and pressure were the outputs. The springboard of setting the three-dimensional size of the organic linker as input data was that it can partly reflect the pore size or topology of the structure, while the polarizability of the organic linker was used to recognize the adsorbent–adsorbate interaction. In addition, adsorbent–adsorbate interaction was partly influenced by the property of the metal center, which was described by the electronegativity. Moreover, the value of electronegativity, which points to the electro-withdrawing property, can determine bond length of the metal center and binding site that form the organic linker. The radius of the metal center can give an index to the coordination environment that the metal center can afford for the organic linker. Moreover, the radius of the metal center can to some degree determine the room for noble gas interacting with metal center.

## 4. Conclusions

In this study, a polarizable and transferable model for describing adsorption and separation of Xe/Kr in MOFs was developed. The reliability of the polarizable model was tested by comparison between adsorption isotherms and selectivities generated from simulation and experiment for MOFs, which consist of various metal centers, organic linkers, topologies, and pore size distributions. The RMSE-I values for Xe and Kr adsorption were decreased from 0.013 to 0.010 cm^3^/m^2^ and 0.005 to 0.004 cm^3^/m^2^, respectively. The RMSE-II values for Xe and Kr adsorption were reduced from 28% to 19% and 38% to 27%, respectively. In terms of the performance of the polarizable model on selectivity, the RMSE-I value for prediction of selectivity was reduced from 2.75 to 1.06, while the RMSE-II value was reduced from 26% to 18% compared with traditional models.

Based on the polarizable and transferable model, high-throughput screening for SP value of Xe/Kr in MOFs was performed in order to design a BPNN model for the prediction of the SP value of Xe/Kr in MOFs based on the physical parameters of the metal center (electronegativity and radius) and the organic linker (three-dimensional size and polarizability). An optimal BPNN model (called BPNN-SP) was constructed through various trial and error tests. Regression coefficient values of the BPNN-SP model for all data sets are higher than 0.995, while values of MAE, MBE, and RMSE are only 0.331, −0.002, and 0.505 mmol/g. About 94.3% of errors stay inside the range of −0.984 mmol/g to 0.984 mmol/g. It should be highlighted that the range of SP values for tested MOFs is from 0 to 42.5 mmol/g. Such a model with high accuracy was then validated by experiment data from six MOFs. The data suggest that the BPNN-SP model tested in this study could be a reliable precursor-based model for predicting the SP value of Xe/Kr mixture in MOFs at various temperatures and pressures.

## Figures and Tables

**Table 1:** RMSE-I and RMSE-II values for Xe adsorption in each MOF calculated from model with and without polarization consideration. (np: model without polarization, p: model with polarization.)

	RMSE-I (cm^3^/m^2^)		RMSE-II (%)	
	np	p	np	p
Mg-MOF-74	0.007	0.004	18	10
Co-MOF-74	0.012	0.016	30	18
Ni-MOF-74	0.023	0.006	47	24
Zn-MOF-74	0.002	0.009	9	14
Cu-BTC	0.013	0.006	27	13
SBMOF-1	0.031	0.030	23	23
IRMOF-1	0.002	0.001	42	29
Average	0.013	0.010	28	19

**Table 2:** RMSE-I and RMSE-II values for Kr adsorption in each MOF calculated from model with and without polarization consideration. (np: model without polarization, p: model with polarization.)

	RMSE-I (cm^3^/m^2^)		RMSE-II (%)	
	np	p	np	p
Mg-MOF-74	0.003	0.001	37	23
Co-MOF-74	0.004	0.004	40	24
Ni-MOF-74	0.008	0.002	60	29
Zn-MOF-74	0.003	0.001	33	22
Cu-BTC	0.001	0.001	15	19
SBMOF-1	0.016	0.018	14	16
IRMOF-1	0.001	0.001	65	54
Average	0.005	0.004	38	27

**Table 3:** Selectivities values from IAST based on experiment data, GCMC based on model without and with polarization consideration, and corresponding RMSE values. (np: model without polarization, p: model with polarization.)

	IAST	np	p
Co-MOF-74	12.44	8.09	9.53
Mg-MOF-74	6.68	7.02	7.68
Ni-MOF-74	10.17	6.36	7.28
Zn-MOF-74	6.25	6.99	7.07
Cu-BTC	8.98	5.51	7.58
IRMOF-1	3.14	2.58	3.01
SBMOF-1	14.56	12.02	11.92
RMSE-I		2.75	1.06
RMSE-II		26%	18%

**Table 4:** Network architectures for prediction of SP and errors associated with each architecture.

Model	Network Architecture	Error Types (mmol/g)			Regression Coefficient			
		MAE	MBE	RMSE	Training	Validation	Testing	All Data
1	5-1	1.277	0.249	1.813	0.975	0.966	0.971	0.973
2	10-1	1.034	0.008	1.594	0.979	0.978	0.977	0.979
3	20-1	0.643	−0.055	0.966	0.995	0.985	0.987	0.992
4	30-1	0.568	−0.059	0.905	0.996	0.988	0.985	0.993
5	40-1	0.697	−0.018	1.100	0.992	0.986	0.983	0.990
6	50-1	0.623	−0.008	0.997	0.995	0.984	0.984	0.992
7	60-1	0.656	0.085	1.193	0.994	0.976	0.974	0.988
8	5-2-1	1.096	0.024	1.579	0.983	0.971	0.970	0.979
9	5-5-1	0.938	0.064	1.377	0.985	0.985	0.982	0.984
10	10-2-1	0.812	−0.029	1.195	0.990	0.987	0.977	0.988
11	10-5-1	0.555	0.036	0.811	0.995	0.992	0.994	0.995
12	10-10-1	0.440	0.000	0.701	0.997	0.993	0.993	0.996
13	20-5-1	0.499	0.041	0.768	0.997	0.992	0.992	0.995
14 (BPNN-SP)	20-10-1	0.331	−0.002	0.505	0.999	0.996	0.995	0.998
15	20-20-1	0.356	0.002	0.620	0.999	0.992	0.992	0.997
16	30-10-1	0.345	0.003	0.648	0.999	0.990	0.992	0.997
17	30-20-1	0.575	−0.001	0.973	0.996	0.981	0.988	0.992
18	30-30-1	0.755	−0.314	1.119	0.992	0.986	0.988	0.990
19	40-10-1	0.432	0.039	0.812	0.999	0.986	0.983	0.995
20	40-20-1	0.542	−0.032	0.927	0.997	0.986	0.984	0.993
21	40-30-1	0.445	−0.052	0.845	0.998	0.988	0.985	0.994
22	40-40-1	0.597	0.007	0.961	0.996	0.986	0.982	0.992

**Table 5:** Comparison between SP values calculated from experiment data and the BPNN-SP model.

MOFs	Temperature (K)	Pressure (Bar)	SP_EXP (mmol/g)	SP_BPNN (mmol/g)
NOTT-100	292	0.1	0.820	1.295
NOTT-100	292	0.4	3.013	3.179
NOTT-100	292	1	6.380	6.387
NOTT-101	292	0.1	0.388	0.460
NOTT-101	292	0.4	1.463	1.289
NOTT-101	292	1	3.401	3.046
NOTT-102	292	0.1	0.179	0.261
NOTT-102	292	0.4	0.632	0.517
NOTT-102	292	1	1.403	1.522
NOTT-103	292	0.1	0.311	0.337
NOTT-103	292	0.4	1.207	0.823
NOTT-103	292	1	2.873	2.961
PCN-14	292	0.1	0.335	0.683
PCN-14	292	0.4	2.366	2.158
PCN-14	292	1	5.461	5.446
UiO-66	298	0.1	0.345	0.884
UiO-66	298	0.4	1.163	1.300
UiO-66	298	1	2.227	2.069
RMSE				0.248

## Data Availability

Data is contained within the article and Supplementary Materials.

## Supplementary Materials

The following supporting information can be downloaded at: https://www.mdpi.com/article/10.3390/molecules28217367/s1, Table S1: LJ parameters for atoms of each adsorbate and adsorbent.
