# Supplementary material for: Prediction of Xe/Kr Separation in Metal-Organic Frameworks by a Precursor-Based Neural Network Synergistic with a Polarizable Adsorbate Model
Source: Molecules. 2023 Oct 31;28(21):7367. doi: 10.3390/molecules28217367 (PMC10648455; doi:10.3390/molecules28217367)
Supplement: Supplementary file 1 [file molecules-28-07367-s001.zip › Supplementary Materials S2.pdf]

Table S1. LJ parameters for atoms of each adsorbate and adsorbent

Atom types	σ(Å)	ε/k_b(K)
C	3.47	47.86
H	2.85	7.65
O	3.03	48.16
N	3.26	38.95
Mg	2.69	55.86
Ca	3.03	119.77
Ti	2.83	8.55
V	2.80	8.05
Mn	2.64	6.54
Fe	2.59	6.54
Co	2.56	7.04
Ni	2.52	7.55
Cu	3.11	2.52
Zn	2.46	62.40
Cd	2.54	114.74
Xe	4.10	221.00
Kr	3.64	166.40
